# Acceptability and Accuracy of Cervical Cancer Screening Using a Self-Collected Tampon for HPV Messenger-RNA Testing among HIV-Infected Women in South Africa

**DOI:** 10.1371/journal.pone.0137299

**Published:** 2015-09-02

**Authors:** Paul C. Adamson, Megan J. Huchko, Alison M. Moss, Hans F. Kinkel, Andrew Medina-Marino

**Affiliations:** 1 School of Medicine, University of California San Francisco, San Francisco, California, United States of America; 2 Research Unit, Foundation for Professional Development, Pretoria, Gauteng, South Africa; 3 Department of Obstetrics, Gynecology, and Reproductive Sciences, School of Medicine, University of California San Francisco, San Francisco, California, United States of America; 4 Drs. Martin and Partners, Edenvale, Gauteng, South Africa; 5 Department of Family Medicine, University of Pretoria, Pretoria, Gauteng, South Africa; 6 School of Health Systems and Public Health, University of Pretoria, Pretoria, Gauteng, South Africa; Georgetown University, UNITED STATES

## Abstract

**Background:**

HIV increases women’s risk for high-risk human papillomavirus (hrHPV) infection and invasive cervical cancer. South Africa has a high HIV prevalence but low cervical cancer screening coverage. Self-collection of cervical specimens and hrHPV testing, including hrHPV messenger-RNA (mRNA) testing, are methods aimed at increasing screening rates. However, data are limited on the acceptability and accuracy of tampon-based self-collection for hrHPV mRNA testing in HIV-infected women.

**Methods:**

We recruited 325 HIV-infected women seeking care at a government HIV clinic in Pretoria, South Africa. A clinician performed a pelvic examination and obtained an endocervical specimen. Study participants performed self-collection using a tampon. Both clinician- and self-collected specimens were tested for hrHPV mRNA. Acceptability of both collection methods was assessed, the prevalence of hrHPV mRNA in our study population was estimated, test positivity of the two collection methods were compared, and test agreement was assessed by calculating the κ-statistic, sensitivity, and specificity.

**Results:**

Over 90% of women reported no difficulties self-collecting specimens and 82% were willing to perform the tampon-collection at home. Based on clinician-collection specimens, the prevalence of hrHPV mRNA in our study population was 36.7% (95% CI: 31.4%– 42.0%). There was no difference in test positivity between clinician-collection, 36.7%, and tampon-collection, 43.5% (p-value = 0.08). Using clinician-collection as the reference test, the sensitivity and specificity for hrHPV mRNA of tampon-collection were 77.4% (95% CI: 69.8–85.0%) and 77.8% (95% CI: 71.9–83.6%), respectively.

**Conclusions:**

Tampon-based self-collection is acceptable to women and has similar hrHPV mRNA positivity rates as clinician-collection, but has reduced sensitivity and specificity compared to clinician-collection. The hrHPV mRNA prevalence in our study population is high, but similar to other high-risk populations, and highlights the need for improved cervical cancer screening. Further research into the optimal use of tampon-based collection as a cervical cancer screening tool is warranted.

## Introduction

Cervical cancer is caused by persistent infection with high-risk human papillomavirus (hrHPV). [1] Globally, cervical cancer leads to approximately 266,000 deaths every year, with over 85% of these occurring in low-resource countries. [2] Many developed countries have greatly decreased the burden of cervical cancer through expansive cervical cytology screening programs utilizing Papanicolaou (Pap) smears. However, cytology-based screening programs in low-resource countries have not had the same success in decreasing cervical cancer burden due to poor organization of government screening programs, low screening coverage, and inadequate quality assurance of screening tests. [3] In South Africa, cervical cancer is the leading cause of cancer-related death among women. [2] South Africa also has the highest burden of HIV in world, with an estimated 6.3 million people living with HIV/AIDS. [4] HIV-infected women are at increased risk for persistent hrHPV infections and, consequently, have significantly greater incidence of invasive cervical cancer. [5] According to the South African national screening guidelines, HIV-negative women should have a Pap smear every ten years starting at age 30 while HIV-infected women should have a Pap smear every three years after being diagnosed with HIV infection. [6,7] The uptake of cervical cancer screening in South Africa is low; in 2013 the screening coverage was estimated to be 54% nationally, with provinces ranging from 32% to 75%. [8] Recently, clinical audits done in four government HIV clinics in Tshwane district, Gauteng Province showed that less than 10% of HIV-infected women had documented cervical cancer screening results in their medical chart. [9] The low screening coverage coupled with a large population of people living with HIV highlight the urgent need to rapidly expand cervical cancer screening services in the country.

Screening for hrHPV using self-collected specimens has been suggested as one way to increase cervical cancer screening coverage in low-resource settings. [10] Some advantages of hrHPV testing as a primary screening tool are that it can be tested by high-throughput laboratory processing with built-in quality control measures, can be used to triage women at higher-risk for developing cervical cancer, and provides a dichotomous result for clinicians [10,11]. Testing self-collected specimens can decrease the burden on both clinics and women, as fewer women must travel to the clinic and book an appointment for a speculum examination by a provider. [10,11] Several studies for HPV DNA testing in low-resource settings have shown that self-collected specimens compare favorably to clinician-collected specimens, with only a small decrease in sensitivity. [12–15] However, hrHPV DNA testing is not an ideal screening tool among HIV-infected women in South Africa because of the high prevalence of hrHPV DNA (46–63%) [16–18]. The high prevalence of hrHPV DNA might lead to decreased specificity of test results, unnecessary invasive procedures, and an overburdening of the referral system.

Molecular diagnostics aimed at detecting hrHPV viral integration, via the production of oncogenic E6 and E7 messenger-RNA (mRNA), might offer an improvement in specificity for predicting precursors to cervical cancer. Initial studies in routine-screening populations reveal a sensitivity and specificity for detecting cervical intraepithelial neoplasia of grade 2 or higher (CIN 2+), to be 94.2–97.5% and 90.2–94.5%, respectively. [19,20]. When compared to HPV DNA tests, the hrHPV mRNA test offers similar sensitivity, but with increased specificity for detecting cervical intraepithelial neoplasia. In populations with a high prevalence of HPV infection, such as HIV-infected women in South Africa, an mRNA-based HPV test has the potential to be a better primary screening tool. Testing for hrHPV mRNA has not yet been implemented as a screening strategy in any low or middle-income countries, and little data exist about the most appropriate methods for specimen collection, including the option of self-collected specimens. Only two studies, one amongst female sex workers in Kenya and another one amongst rural women in Nepal, have evaluated the use of self-collected swabs for hrHPV mRNA testing in low-resource settings. [21,22] Self-collection using tampons might provide another option for screening programs, as tampons are inexpensive, widely available, and have been show to be an acceptable collection method among women in South Africa. [23] To date, there are no studies evaluating the performance and acceptability of tampon-based self-collection for hrHPV mRNA testing and no data about the prevalence of hrHPV mRNA in HIV-infected women.

Our objectives were to 1) estimate the prevalence of hrHPV mRNA among a cohort of HIV-infected women, 2) compare the test positivity between the two collection methods, 3) assess the accuracy and agreement of self-collected tampons compared to clinician-collected specimens for hrHPV mRNA testing, and 4) assess the acceptability of the self-collected tampon method.

## Materials and Methods

### Study Population

We conducted a cross-sectional study from February 2014 through April 2014. We enrolled 325 HIV-infected women seeking care at a government HIV clinic in Tshwane District, Gauteng Province, South Africa.

Inclusion criteria were HIV-infected women, 25 years or older, who did not have a cervical cytology test result documented in their chart within the past three years. Women who had a hysterectomy or who were currently menstruating were excluded from the study. The women who were menstruating were invited to come back to the clinic for study enrollment after menstruation.

Women who were eligible to participate were seen by a study nurse who obtained informed consent. Participants then were administered a brief questionnaire to collect socio-demographic, reproductive, and health data, as well as an assessment of knowledge regarding cervical cancer. HIV viral load and CD4+ cell count were extracted from the patient’s medical record.

### Study Visit and Sample Collection

After administering the intake questionnaire, the study nurse performed a pelvic examination. Lubricant was not applied to the speculum prior to insertion. After speculum placement, the cervical os was visualized and a cotton-tipped swab was used to remove any excess secretions. The cervical specimen was collected by inserting the broom-like collection device into the cervical os and rotating five times. The collection device was then immediately swirled into a ThinPrep Pap collection container containing PreservCyt solution (Hologic Incorporated, Bedford, MA) to dislodge cervical cells. The collection brush was then discarded.

After obtaining the cervical specimen, the study nurse instructed the study participant on how to perform the tampon-based self-collection. Briefly, the woman was instructed to insert the tampon into her vagina, leave it in-place for one to two hours, then to remove and place the tampon into the provided collection tube and return it to the study nurse. Tampon-collection was performed using mini-sized tampons, with light absorpency (Lil-Lets Brand, Westville, South Africa). Tampon insertion and removal was not witnessed by the study nurse. The tampon collection tube was a 30mL plastic bottle that contained 10mL of saline solution to prevent drying of the sample. At the time of receipt, the nurse verified that the tampon was completely submerged into the saline and the collection bottle was closed correctly.

After returning the tampon specimen, a second nurse administered an acceptability questionnaire about the woman’s experiences with the pelvic exam and the tampon-based self-collection. To minimize bias, the study nurse who performed the pelvic examination was in another room and did not participate in the post-test questionnaire administration. The questionnaire used a Likert scale, from 1 (most favorable) to 5 (least favorable), to evaluate experiences related to perception of care, comfort, privacy, embarrassment, and pain for both collection methods individually. The questionnaire also assessed difficulties encountered during tampon-collection and had questions regarding the ability to perform self-collection, including home collection.

### Specimen Handling, Transport and Testing

The ThinPrep collection containers and the tampon collection tubes were stored in a refrigerator (4–8°C) within an hour after collection. The specimens were transported three times per week (usually Monday, Wednesday, and Friday) in a cold box for processing and testing by Toga Labs (South African National Accreditation Service # M0542, Harmelia, Gauteng, South Africa).

#### Cytology

Cytological smears, from clinician-collected specimens only, were prepared on the ThinPrep T2000 slide preparation machine and were read by two cytotechnologists blinded to any results from molecular testing. Smears were classified according to the 2001 Bethesda System for cervical cytology. [24] Study participants were notified of their cytology results within three weeks of their screening visit and a copy of their results was included in the clinic-based medical chart for future reference.

Women were referred for colposcopy if they had abnormal cytology results, including low-grade intraepithelial lesions (LSIL), atypical squamous cells cannot exclude high-grade lesion (ASC-H) and high-grade intraepithelial lesions (HSIL). Women with atypical squamous cells of undetermined significance (ASC-US) were instructed to repeat cytology test in six months. Women with normal cytology results were instructed to follow routine screening recommendations.

#### hrHPV mRNA Testing

hrHPV mRNA testing was performed on both the clinician- and self-collected specimens. For the clinician-collected specimens, a 1mL aliquot was transferred from the ThinPrep Pap Test container to an Aptima Specimen Transfer tube within one week of receipt at the laboratory. These specimens were then batched and tested using the Aptima HPV assay, according to manufacturer’s instructions. The Aptima HPV assay detects E6/E7 mRNA of 14 high-risk HPV types (16, 18, 31, 33, 35, 39, 45, 51, 52, 56, 58, 59, 66, and 68). [25] Each testing assay utilized an internal control, which monitors target capture, amplification, and detects assay steps, but is measured separately from the HPV signal by separate probes and light emission. Test results were either positive, negative, or invalid. Invalid results were generated if the internal control surpassed a signal cutoff threshold, as set by the manufacturer. [25] Amplification inhibitors contaminating a specimen or the presence of a precipitate might trigger an invalid result. All invalid results were repeated; results that were invalid on two separate testing runs were reported as invalid. Results were shared with the clinic staff. Women with a normal cytology test, but a positive hrHPV mRNA result were recommended by the study team to seek further follow-up at the clinic.

Upon delivery to the laboratory, the tampon specimens were removed from the collection container and the liquid was extracted from the tampon. This liquid was spun down to make a cell pellet. The supertanant was aspirated until 2ml of the liquid remained, at which point the cell pellet was resuspended. Then 1ml of this cell-containing liquid was extracted into an Aptima Specimen Transport Tube and the tampons were discarded; the remaining 1mL of liquid was stored in a -80°C freezer. The specimens in the Aptima Specimen Transfer Tube were tested on the Aptima Assay, according to the manufacturer’s instructions.

### Sample Size

In order to estimate the hrHPV mRNA positivity in our study population, we used published test characteristics and the prevalence of cervical dysplasia as reported in a recent study within an HIV-infected population in South Africa. [19,20,26,27] Based on these assumptions, we estimated the prevalence of mRNA-positive test results to be 32% in the clinician-collected arm. We conducted a non-inferiority comparison and our hypothesis was that the difference in hrHPV mRNA positivity between the tampon-based self-collection and clinician-collection methods would be less than 10%. Using an α = 0.05 and β = 0.80, we needed 309 study participants to detect at least a 10% difference in HPV positivity.

### Statistical Analysis

Acceptability of the Pap test and the tampon self-collection were assessed using a Likert scale based on different acceptability categories (e.g.–embarrassment, privacy, pain). The mean and standard deviation for Likert scale data were calculated for each category. The mean from each collection method were compared using a student t-test. We compared the overall prevalence of hrHPV mRNA between the two collection methods. The clinician-collected hrHPV mRNA test results were used as the reference standard to estimate the sensitivity and specificity, with corresponding 95% confidence intervals, of the tampon-collection method. We calculated the k-statistic, with corresponding 95% confidence intervals, to assess the agreement between the two collection methods. All statistical analyses were performed in Stata (StataCorp, College Station, TX).

### Ethics

All study procedures were explained to participants and written informed consent was obtained. All study protocols and documents were reviewed and approved by the institutional review boards at the University of Pretoria, Pretoria, South Africa (#112/2012) and the University of California, San Francisco, San Francisco, CA (#13–11129).

## Results

### Socio-demographic and Clinical Characteristics

**Fig 1:**
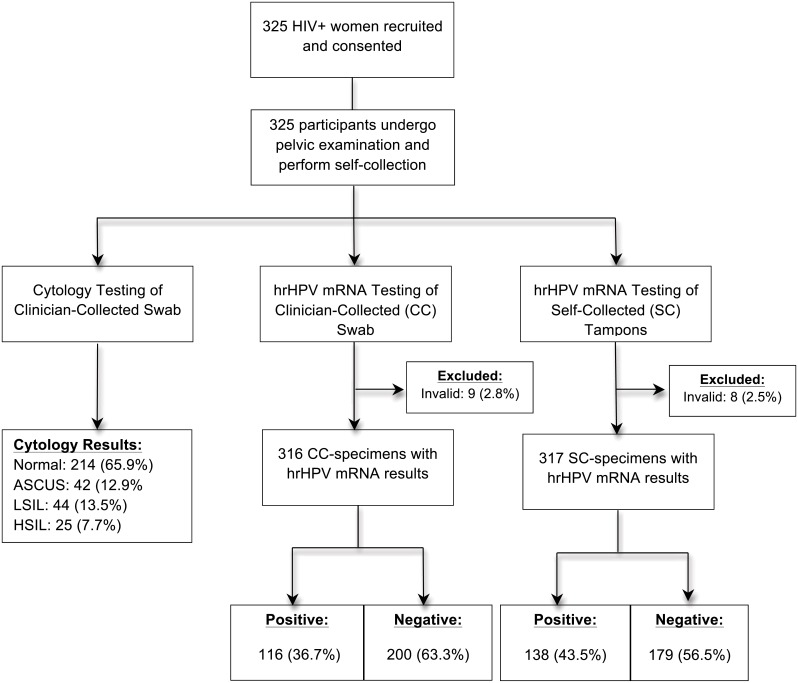
Flow diagram of study recruitment, specimen collection, laboratory testing, and test results.

In total, 325 women consented and participated in study procedures (Fig 1). The median age of our study population was 41.6 years (IQR: 34.9–47.5) and 57.5% (n = 187) of the women were single or never married (Table 1). The median CD4+ cell count among study participants was 496 cells/mL and 85.7% (n = 263) had an undetectable HIV viral load. There were 110 women (34.1%) with abnormal results on their baseline cytology specimens. In total, 176 women (54.0%) reported never hearing of cervical cancer before and 115 women (35.4%) reported ever being screened for cervical cancer.

**Table 1:** Demographic and Clinical Characteristics of 325 HIV-Infected Women in Pretoria, South Africa.

Demographics	N = 325
	n (%) or median (IQR)
Age (years)	41.6 (34.9–47.5)
Monthly Income (ZAR^a^)	1700 / (1000–3000)
Education	
None	15 (4.6)
Incomplete up to 12th Grade	206 (63.4)
Matric / Completed 12^th^ Grade	102 (31.4)
Higher Education	2 (0.6)
Marital Status	
Single	187 (57.5)
Married	75 (23.1)
Widowed/Separated/Divorced	63 (19.4)
Smoking^b^	
Current Smoker	11 (3.4)
Ever Smoker	47 (14.6)
Pack Years	0.55 (0.2–1.2)
Currently Using Birth Control	141 (43.3)
Birth Control Method	
Condoms	77 (23.7)
Hormonal	56 (17.2)
IUD	4 (1.2)
Other	21 (6.5)
Ever Screened for Cervical Cancer	115 (35.4)
Ever Heard About Cervical Cancer	168 (54.0)
**Clinical Characteristics**	
Most Recent CD4 Count (cells/μL)	496 (328–649)
CD4 Checked in Last Year	196 (60.3)
Most Recent Viral Load (copies/mL)	1644 (443–14, 890)
Undetectable HIV Viral Load	263 (85.7)
Viral Load Check in Last Year	240 (73.8)
Cytology Result	
Negative	214 (65.9)
ASCUS	42 (12.9)
LSIL	44 (13.5)
HSIL	25 (7.7)
hrHPV mRNA positive (clinician-collected)^c^	116 (36.7)*
hrHPV mRNA positive (self-collected)^d^	138 (43.5)*

^a^ South African Rand

^b^ Current Smoker = if participant currently reports smoking cigarettes; Ever Smoker = if participant reported any history of smoking cigarettes.

^c^ Excluding 9 specimens (n = 316)

^d^ Excluding 8 specimens (n = 317)

* P-value = 0.08

### hrHPV Laboratory Results

All 325 women had paired clinician-collected and self-collected specimens obtained for laboratory testing. Nine results (2.8%) were excluded among the clinician-collected specimens and eight (2.4%) among the tampon specimens due to laboratory processing errors or invalid hrHPV mRNA results.

Of the 316 clinician-collected specimens included in the analysis, 116 tested positive for hrHPV mRNA, corresponding to a prevalence of 36.7% (95% CI: 31.4%– 42.0%) (Table 1). The test positivity rate for hrHPV mRNA in self-collected specimens was 43.5% (n = 138; 95% CI: 38.0%- 49.0%). There was no difference in the rate of test positivity for hrHPV mRNA between clinician-collected and self-collected specimens (36.7% vs. 43.5%; p-value = 0.08).

Results from each of the collection method are presented in Table 2. The percent agreement between the two collection methods was 77.6% and the κ-statistic was 0.54 (95% CI: 0.44–0.63). Using clinician-collected specimens as the reference test, the sensitivity and specificity of the self-collected tampons were 77.4% (95% CI: 69.8%- 85.0%) and 77.7% (95% CI: 71.9%- 83.6%), respectively.

**Table 2:** Two-by-Two Table of Self-Collected Specimens Compared to Clinician-Collected Specimens for hrHPV mRNA Testing.

	**Clinician-Collected Specimens**		
	Positive	Negative	Total
**Self-Collected Tampon**			
Positive	89	43	132
Negative	26	150	176
Total	115	193	308*
	**Estimate**	**95% CI**	
**Sensitivity**	77.4%	69.8%- 85.0%	
**Specificity**	77.7%	71.9%- 83.6%	
**κ-statistic**	0.54	0.44–0.63	

*Excluding results in nine clinician-collected specimens and in eight self-collected specimens

### Acceptability of Collection Methods

Participants reported feeling slightly better cared for during the clinician-collection (mean = 1.04) compared to self-collection (mean = 1.14; p-value = <0.001), but there were no other significant differences in privacy, embarrassment, discomfort or pain between the two collection methods (Table 3). There were 147 women (45.2%) who were already familiar with using tampons and 291women (89.5%) reported that the instructions prepared them well for the tampon collection. Twenty-eight women (8.6%) reported difficulties with performing the self-collection. A total of 266 women (81.8%) reported that they would be willing to perform tampon-collection at home and bring the specimen with them to clinic. When asked to choose one collection method, 178 women (54.8%) responded that they would prefer the clinician-collection method. However, when given the options of 1) performing self-collection at home, 2) performing self-collection in the clinic, or 3) undergoing clinician-collection, 44 women (10.1%) preferred home-based self-collection, 211 (64.9%) preferred clinic-based self-collection, and 49 (15.1%) preferred clinician-collection (n = 21 missing values; 6.5%). These results on preference present conflicting data and do not to meet our internal validity standard, as the questions were not adequately piloted prior to usage. The data were excluded from further analysis.

**Table 3:** Acceptability of Tampon-Collection Compared to Clinician-Collection for hrHPV mRNA Testing.

	Tampon—Collection	Clinician-Collection	
Acceptability Measure*	Mean (SD)	Mean (SD)	p-value
How well cared for did you feel?	1.14 (0.44)	1.04 (0.27)	<0.001
How well was your privacy handled during the test?	1.01 (0.10)	1.02 (0.27)	0.62
Did you feel embarrassed?	1.09 (0.48)	1.13 (0.45)	0.17
Did the test cause you any genital discomfort?	1.35 (0.62)	1.32 (0.58)	0.54
Did the test cause you any genital pain?	1.14 (0.38)	1.17 (0.41)	0.36

*Range from 1 to 5, 1 = most favorable and 5 = least favorable

## Discussion

To our knowledge, this is the first report of hrHPV mRNA prevalence within a primary screening population infected with HIV. Based on clinician-collection, we report the prevalence of hrHPV mRNA to be 36.7%. There are only two reports on the prevalence of hrHPV mRNA in low-resource settings. [21,22] The prevalence in our primary screening study population of HIV-infected women is slightly higher than the reported prevalence from a cohort of female sex-workers in Kenya (30%) and much higher than a cohort of women undergoing screening at a health camp in rural Nepal (9.6%). [21,22] As expected, the prevalence of hrHPV mRNA in our study population is lower than estimates of hrHPV DNA prevalence in HIV-infected women in South Africa (46–68%). [17,18,27,28] Not all women who have been infected with hrHPV, as measured by the DNA test, produce E6/E7 mRNA, which is the result of viral integration into host DNA. Consequently, the hrHPV mRNA test might be a more specific primary screening tool to detect women at higher risk of developing cervical cancer. Ultimately, the mRNA test may offer greater discriminatory power regarding which women need further screening and treatment in populations with a high prevalence of HPV infection. This would be of particular importance in the allocation and utility of health care resources available in low-resource settings.

We found no difference in the hrHPV mRNA positivity rate between the two collection methods. Furthermore, the κ—statistic (0.54) indicated “moderate” agreement. Few studies have evaluated the performance of hrHPV mRNA testing of self-collected versus clinician-collected specimens, and even fewer studies report using similar study measures. Johnson et al. reported a similar hrHPV mRNA positivity rate between clinician- and self- collected swabs, with a κ—statistic of 0.62, [22] and Chernesky et al. reported 82.0% agreement between the two collection methods with a κ—statistic of 0.63. [22,29] There exist limited data on performance of self-collected specimens for hrHPV mRNA testing for which to compare our results. However, numerous studies have evaluated self-collection for hrHPV DNA testing and are useful to contextualize our results. Our findings comparing positivity rates between the two collection methods and the kappa-statistic are very similar to reports evaluating hrHPV DNA self-collection. [12,14,30–34]

We report that out of 115 hrHPV mRNA-positive clinician-collected specimens, 89 tampon-collected specimens were also positive for hrHPV mRNA, corresponding to a sensitivity of 77.4%. Likewise, of 193 clinician-collected specimens negative for hrHPV mRNA, 150 tampon-collected specimens were also negative for hrHPV mRNA, corresponding to a specificity of 77.7%. Data reporting the sensitivity and specificity of tampon-based self-collection in this way are limited, even in the hrHPV DNA testing literature. Jones et al. compared tampon-based self-collection to clinician-collection using two separate HPV DNA tests. They report a wide range of sensitivities for tampon collection, 59.5% and 91.8% for the two different HPV DNA tests performed, and estimate the specificity to be 86.2% and 90.6%. [14] Our findings are different from those reported by Jones et al., but it is unclear if these differences are due to different duration of collection time, different order of specimen collection, or if they reflect the differences in testing methods. It is important to note that these estimates, in addition to our own, are for comparison of the two collection methods, using clinician-collection as the reference; we did not collect histology data on these women and thus cannot estimate the sensitivity and specificity of tampon-collection for predicting CIN 2+.

One reason for the lower sensitivity of the tampon-collection might be due to sampling location. The clinician-collected specimen, obtained directly from the cervix, preferentially collects cervical cells in the transformation zone, whereas the tampon-method samples a mix of cells from both the cervix and vagina, and therefore might not collect enough cells from the transformation zone. It is possible that extending the time the tampon was held might increase the sensitivity, but might lead to decreased acceptability. [10] The difference in sampling location might also be an explanation for false positive results as the tampon might have picked up vulvovaginal HPV infections, which do not necessarily coincide with cervical infections. [35]

Tampon-based self-collection proved to be an acceptable method of cervical cancer screening within our cohort of HIV-infected women. Very few women reported difficulty performing the tampon collection and nearly all women reported positive experiences with collection. Our findings of tampon acceptability are supported by other research among HIV-positive women in South Africa, suggesting that a large proportion of urban women might actually prefer tampon-based self-collection to other self-collection methods. [23] One advantage of tampon-based self-collection is that many women are already familiar with using tampons (45% in our study population), which might lead to greater preference for tampon-based self-collection over other more unfamiliar methods, such as a brush or swab. Other advantages of tampons are that they are inexpensive and easily accessible. Providing options for self-collection based upon women’s preferences is likely to increase screening coverage, and our data suggest that tampons are an acceptable option.

Our study had several strengths. First, we recruited and screened a large sample of HIV-infected women who represent a high-risk primary screening population. Secondly, both collection methods were performed sequentially on the same day, allowing for direct comparison of the samples collected. Third, we were able to assess both the acceptability as well as the performance of the tampon-collection method for hrHPV mRNA testing. However, our study has limitations. One limitation is that we were not able to directly evaluate which collection method was more favorable. Second, we did not perform cervical biopsies on study participants and do not know the true disease status of these women.

In summary, we found that tampon-based self-collection for hrHPV mRNA could function as a viable method for cervical cancer screening among HIV-infected women in low-resource settings, increasing the options available to women and program planners. The collection method and test might be offered as a way to increase primary screening coverage among women with limited access to screening services or who prefer self-collection methods. In light of our findings, one option to improve the utility of the tampon-based self-collection method and mRNA testing might be to screen more frequently using this method, which might increase the probability of detecting cervical infections. Given the lower specificity of the tampon-based self-collection method, women with positive results would need follow-up with a confirmatory test to reduce the need and costs of unnecessary follow-up by colposcopy. Further research is needed to 1) evaluate the tampon-collection and hrHPV mRNA testing against true cervical disease, 2) explore the implementation and uptake of the collection and testing method in a routine screening population, taking into account women’s preferences for screening methods, and 3) optimize the timeline for testing.
